# Supplementary material for: Structural basis of TMPRSS11D specificity and autocleavage activation
Source: Nat Commun. 2025 May 10;16:4351. doi: 10.1038/s41467-025-59677-3 (PMC12065894; doi:10.1038/s41467-025-59677-3)
Supplement: Supplementary file 1 — Supplementary Information [file 41467_2025_59677_MOESM1_ESM.pdf]

Supplementary Information

Structural basis of TMPRSS11D specificity and autocleavage activation

Bryan J. Fraser*, Ryan P. Wilson, Sára Ferková, Olzhas Ilyassov, Jackie Lac, Aiping Dong, Yen-Yen Li, Alma Seitova, Yanjun Li, Zahra Hejazi, Tristan M.G. Kenney, Linda Z. Penn, Aled Edwards, Richard Leduc, Pierre-Luc Boudreault, Gregg B. Morin*, François Bénard*, and Cheryl H. Arrowsmith*

***Corresponding Authors**

Bryan J. Fraser, Bryanj.fraser@utoronto.ca
Gregg B. Morin, gmorin@bcgsc.ca
François Bénard, fbenard@bccrc.ca
Cheryl H. Arrowsmith, Cheryl.arrowsmith@uhn.ca

Table of contents

Supplementary Figures (1 to 20) p. 2-17

Supplementary Tables (1 to 4) p. 18-19

Supplementary Methods p. 20-27

Supplementary References p. 27

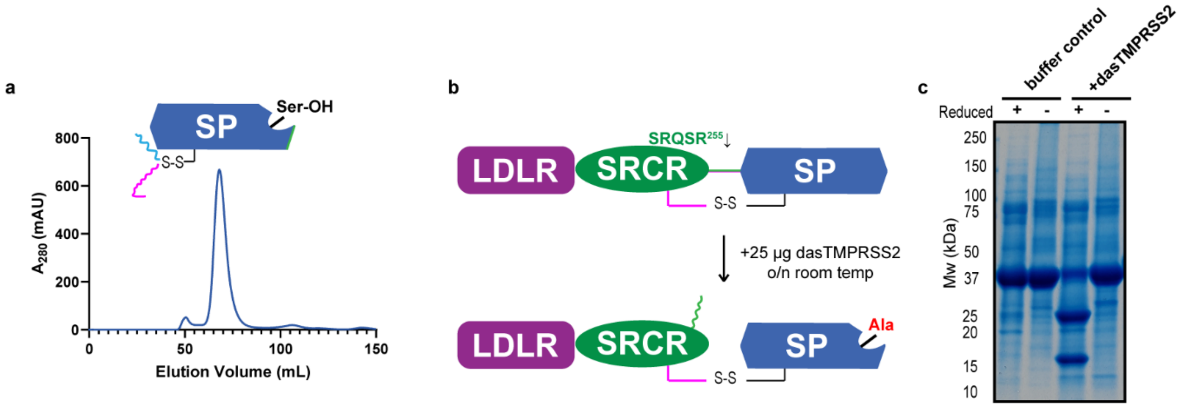

Supplementary Figure 1. Purification of dasTMPRSS11D and demonstration that TMPRSS2 cleaves its own SRQSR255 zymogen activation motif. a, Size-exclusion chromatography purification of active dasTMPRSS11D. Approximately 18 milligrams of crude, active dasTMPRSS11D protein was loaded to a HiLoad 16/60 SuperDex 75 gel filtration column (4 °C, 1 mL/min, 50 mM Tris pH 8.0, 200 mM NaCl). Protein content was monitored by A_{280}. **b,** Schematic of a soluble TMPRSS2 Ser441Ala protein construct before and after treatment with a trace amount of active dasTMPRSS2 protease. **c**, SDS-PAGE analysis of (**b**) for TMPRSS2 Ser441Ala protein incubated overnight with buffer control or 25 µg active dasTMPRSS2 protease. The protein gel image is consistent with results obtained across n=3 independent biological experiments.

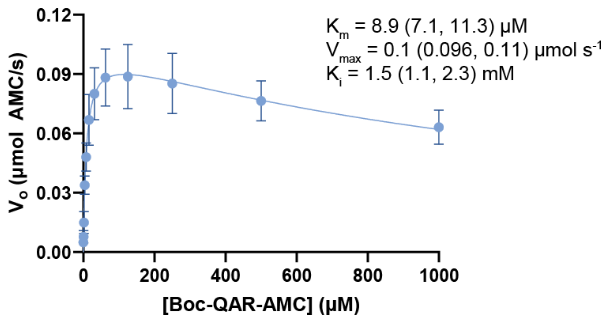

Supplementary Figure 2. Kinetic characterization of Boc-QAR-AMC substrate with dasTMPRSS11D. Michaelis-Menten plot of dasTMPRSS11D (3 nM) with Boc-QAR-AMC substrate. Initial reaction velocity plots were determined by taking the slope across the first 120 seconds of the reaction. Data are shown as mean values +/- SD for experiments performed in technical duplicate across 4 independent biological replicates (total n=8). Michaelis-Menten curves were fit in Graphpad Prism with a substrate inhibition parameter, producing the indicated values for maximum reaction velocity (V_{max}), Michaelis constant (K_m) and substrate apparent inhibition constant (K_i).

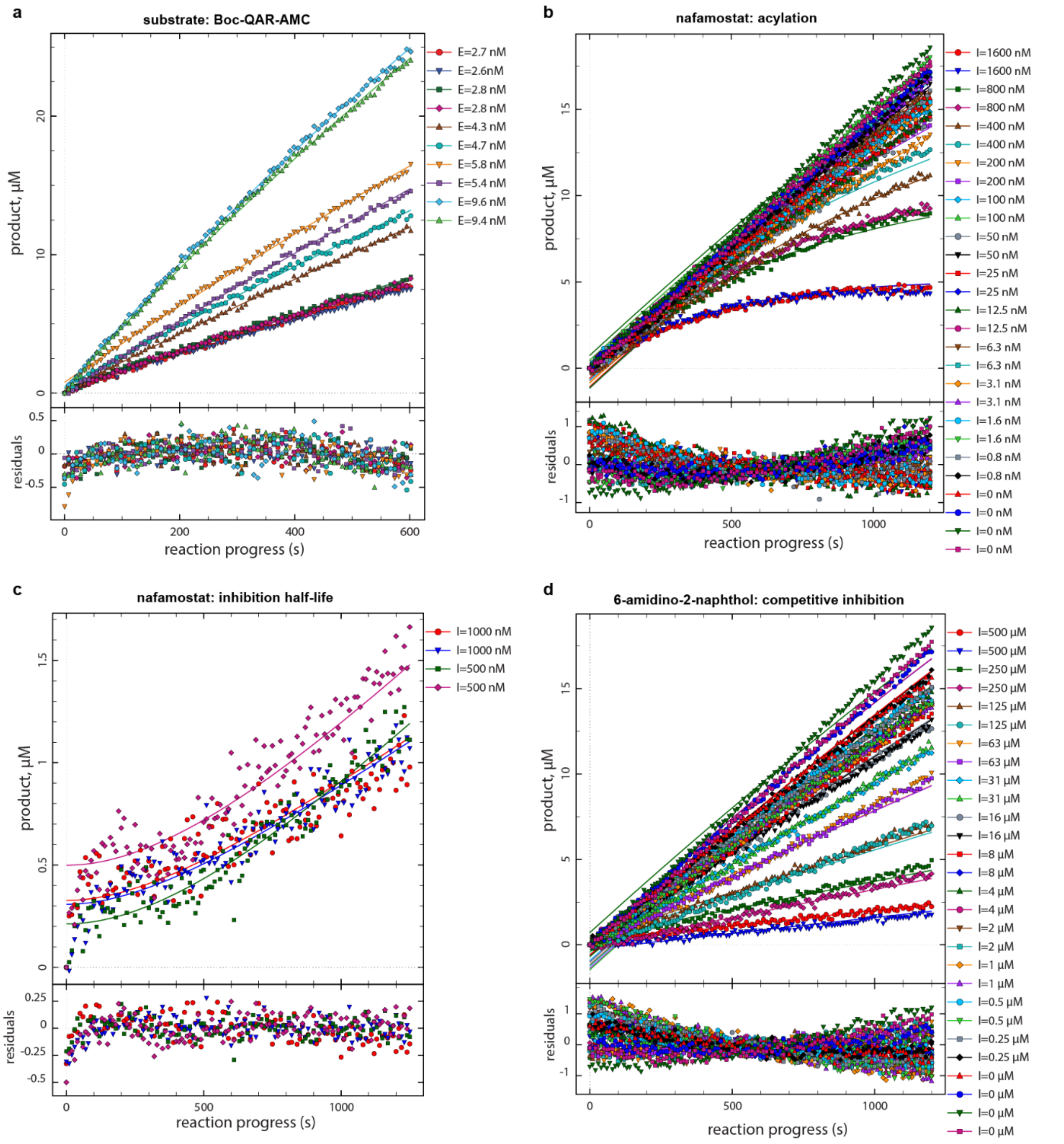

Supplementary Figure 3. TMPRSS11D enzyme kinetic models to determine kinetic inhibition parameters for 6-amidino-2-naphthol and nafamostat. a, TMPRSS11D Boc-QAR-AMC substrate conversion rate, k_{sub}. Reaction progress curves were generated with the indicated concentrations of dasTMPRSS11D enzyme and 100 µM Boc-QAR-AMC substrate and monitored for AMC product formation. Progress curves were fitted across all enzyme concentrations to determine the k_{sub} parameter (Model 1; Methods). Datapoints and curve fits are shown, with data residuals (curve fit value – experimental data) plotted below. **b**, TMPRS11D inactivation potency parameter, k_{inact}/K_I, with nafamostat. Progress curves were generated using 3 nM dasTMPRSS11D, 100 µM Boc-QAR-AMC substrate, and the indicated concentrations of nafamostat. Inhibitor and substrate were simultaneously transferred to wells containing dasTMPRSS11D and fluorescence was monitored immediately. Data curve-fitted according to Model 2 (Methods). **d**, dasTMPRS11D nafamostat inhibition half-life parameter, $t_{1/2}$. Data was generated using 3 nM dasTMPRSS11D and 100 µM Boc-QAR-AMC substrate. Nafamostat (at the indicated concentrations) was incubated with dasTMPRSS11D for 3 minutes prior to substrate transfer. Data was curve-fitted to determine the

hydrolysis rate of the dasTMPRSS11D:nafamostat acyl-enzyme complex (Model 3). **d**, dasTMPRSS11D inhibition constant (K_i) for 6-amidino-2-naphthol. Progress curves were generated using 3 nM dasTMPRSS11D, 100 µM Boc-QAR-AMC substrate in the same assay protocol as (**b**). Data was curve-fitted according to Model 4 (Methods).

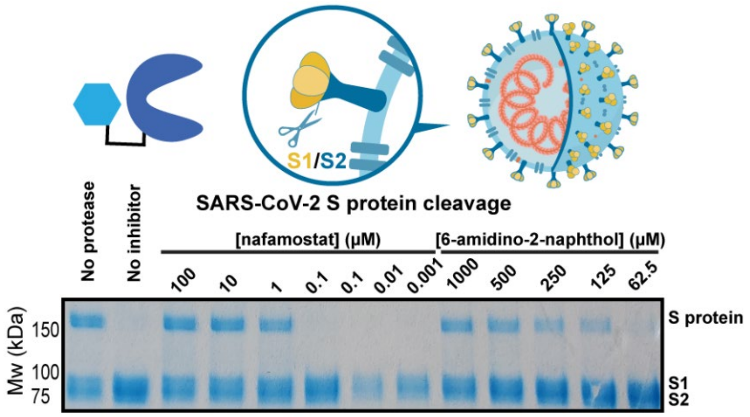

Supplementary Figure 4. The dasTMPRSS11D protease can cleave recombinant SARS-CoV-2 S protein as a substrate and is blocked by small molecule inhibitors. SARS-CoV-2 S protein cleavage assay with dasTMPRSS11D protease and S protein substrate. Purified recombinant S protein was incubated with 10 nM dasTMPRSS11D protease for 15 minutes at 20°C. Samples were prepared for SDS-PAGE through addition of 4X Laemelli buffer and heated (95°C for 5 minutes), then separated on a gel and stained with Coomassie blue. The protein gel image is consistent with results obtained across *n*=3 independent biological experiments.

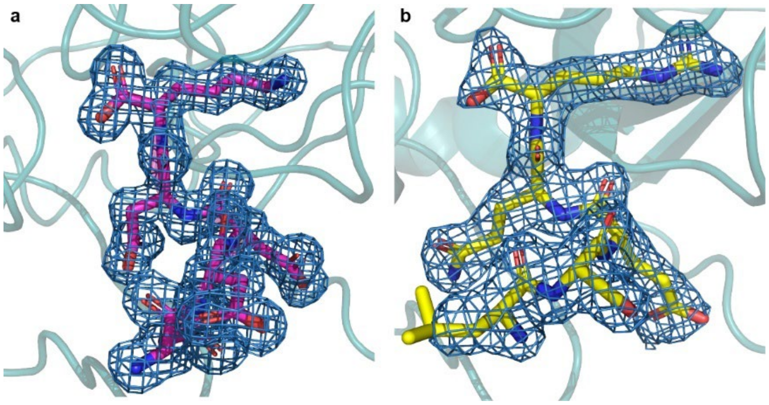

Supplementary Figure 5. Peptide ligand omit maps for TMPRSS11D crystal structures. mFo-DFc omit maps (contoured at 4.0σ) for the DDDDK-CO_2^- (**a**; PDB 8VIS) and LSEQR-CO_2^- (**b**; PDB 9DPF) peptide ligands found occupying the substrate binding cleft of TMPRSS11D.

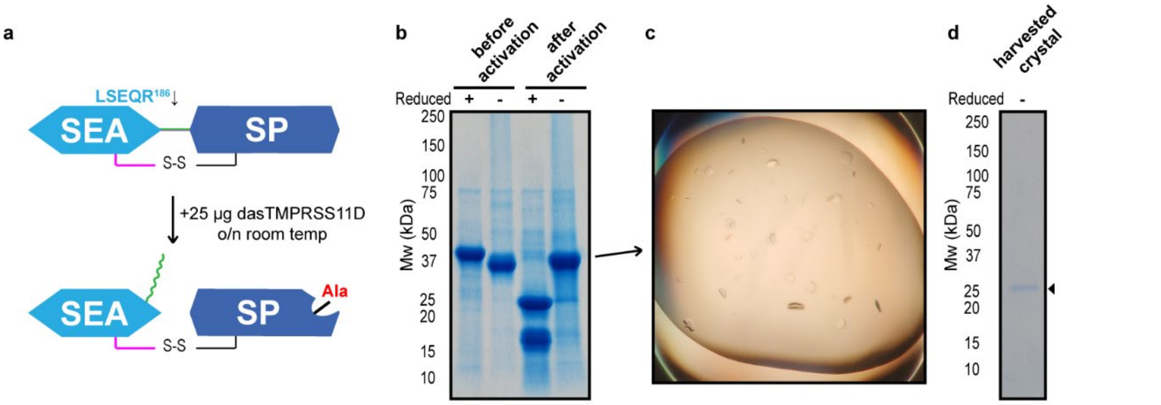

Supplementary Figure 6. The eTMPRSS11D S368A protein crystal is comprised of a protein lacking the SEA domain. a, Schematic of a soluble TMPRSS11D S368A protein construct before and after treatment with a trace amount of active dasTMPRSS11D protease. **b,** Coomassie-stained SDS-PAGE gel of the eTMPRSS11D S368A protein before and after overnight treatment with 25 µg active dasTMPRSS11D protease. **c**, Protein crystals formed for eTMPRSS11D S368A that provided a high resolution TMPRSS11D crystal structure (PDB 9DPF). **d**, Coomassie-stained SDS-PAGE gel of the eTMPRSS11D S368A protein crystals amenable to structure determine. Crystals were harvested by a loop, washed extensively in fresh precipitant buffer, then prepared for SDS-PAGE through addition of 4X Laemelli buffer. The protein gel images are consistent with results obtained across *n*=3 independent biological experiments.

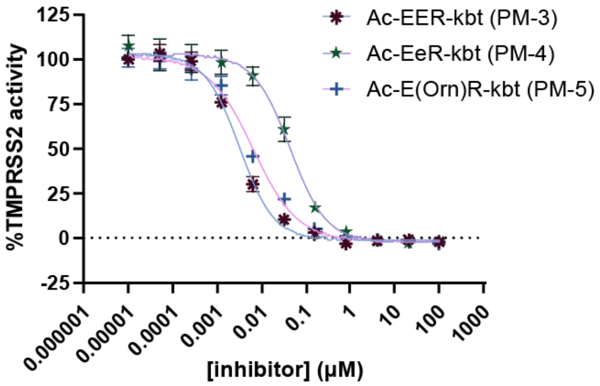

Supplementary Figure 7. Engineered peptidomimetic inhibitors potently inhibit recombinant TMPRSS2 and binding affinity depends on the P2 ligand residue. dasTMPRSS2 (1.5 nM enzyme) IC_{50} plot for ketobenzothiazole (kbt)-containing peptidomimetics 3-5 (PM-3-5). Assays contained a final concentration of 100 µM Boc-QAR-AMC substrate and relative protease activity was determined across the first 60 seconds of the reaction after substrate addition. Data are shown as mean values +/- SD for experiments performed in technical duplicate across 4 independent biological replicates (total *n*=8). PM-3: Ac-Glu-Glu-Arg-kbt; PM-4:Ac-Glu-D-Glu-Arg-kbt; PM-5:Ac-Glu-Orn-Arg-kbt.

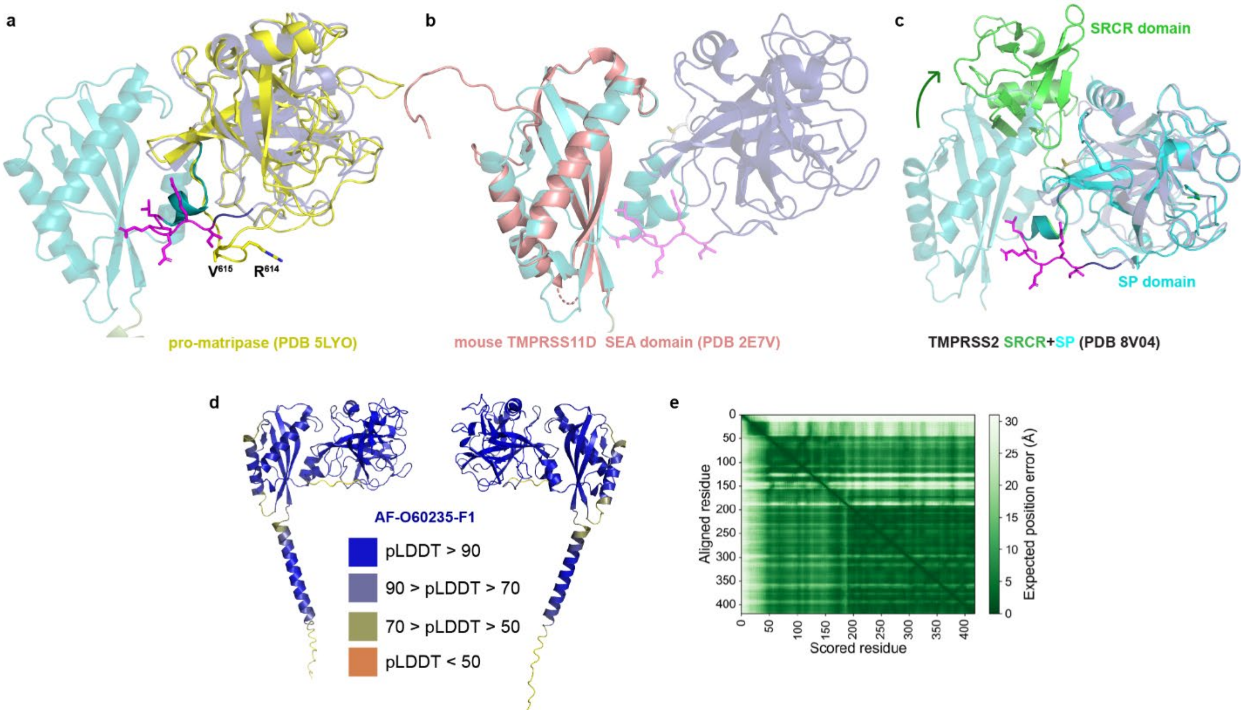

Supplementary Figure 8. The AlphaFold2 TMPRSS11D structure accurately represents the full-length, zymogen form of the protease. a, Structural superposition of the serine protease domain of the AlphaFold2 TMPRSS11D model (dark purple; AF-O60235-F1) with the crystal structure of the pro-matriptase serine protease domain (yellow; PDB 5LYO). The zymogen activation motif of TMPRSS11D is indicated with magenta sticks and the zymogen activation motif of pro-matriptase indicated with yellow sticks. The TMPRSS11D SEA domain is shown as a teal cartoon. **b**, Superposition of mouse TMPRSS11D SEA domain (salmon cartoon; PDB 2E7V) upon the SEA domain of the AlphaFold2 TMPRSS11D structure. **c**, Superposition of the TMPRSS2 serine protease domain (teal; PDB 8V04) upon the serine protease domain of the AlphaFold TMPRSS11D serine protease domain (purple; AF-O60235-F1). The SRCR domain of TMPRSS2 is shown as a green cartoon. The relative positioning of the TMPRSS2 SRCR domain to the TMPRSS11D SEA domain is indicated with a green arrow. **d**, Predicted local distance difference test (pLDDT; colored according to confidence thresholds) and (**e**) Predicted Aligned Error (PAE) of the AF-O60235-F1 structure.

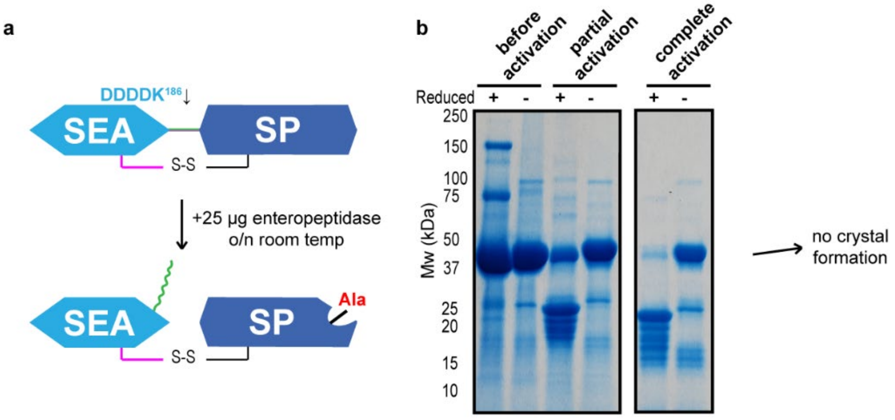

Supplementary Figure 9. The dasTMPRSS11D S368A protein is amenable to overexpression, purification, and zymogen activation but resists crystallization. a, Schematic of a dasTMPRSS11D S368A protein construct

before and after treatment with a trace amount of active human enteropeptidase. **b**, Coomassie-stained SDS-PAGE gel of the dasTMPRSS11D S368A protein before enteropeptidase treatment, 6 hors after enteropeptidase addition (partial cleavage) and 18 hrs after enteropeptidase addition (complete cleavage). Approximately 25 µg active human enteropeptidase was used for dasTMPRSS11D S368A protein cleavage. The protein gel image is consistent with results obtained across *n*=3 independent biological experiments.

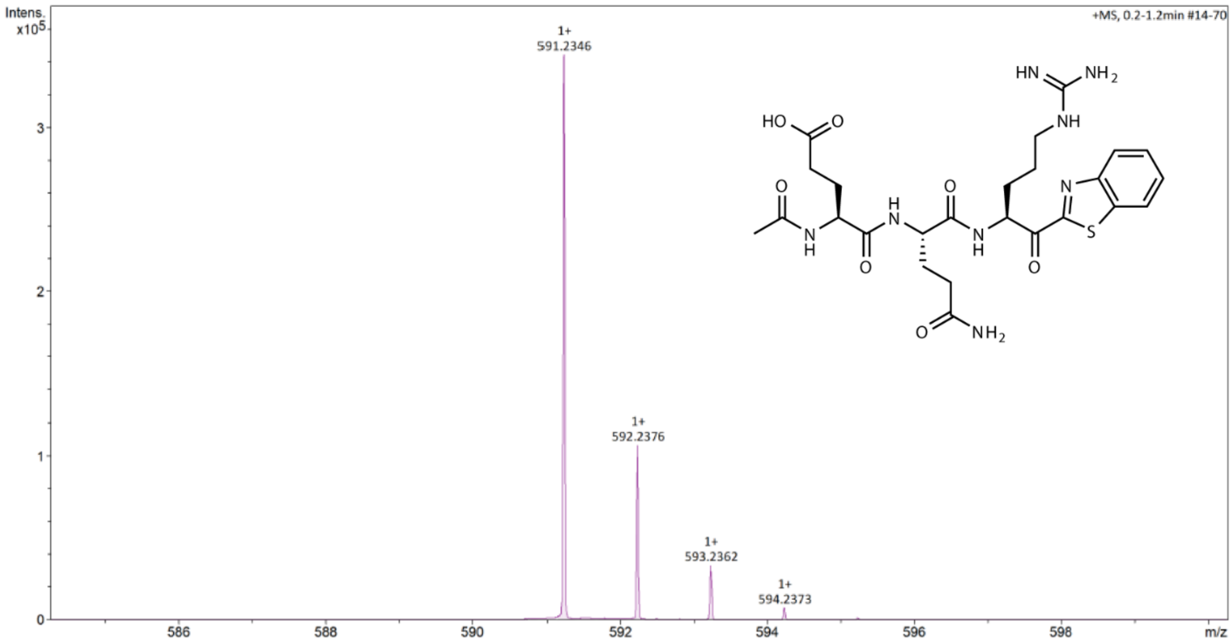

Supplementary Figure 10. Isotopic profile for the most abundant ion (singly charged) peptidomimetic **1**, $[M+H]^+$ detected with high-resolution mass spectrometry (Qtof).

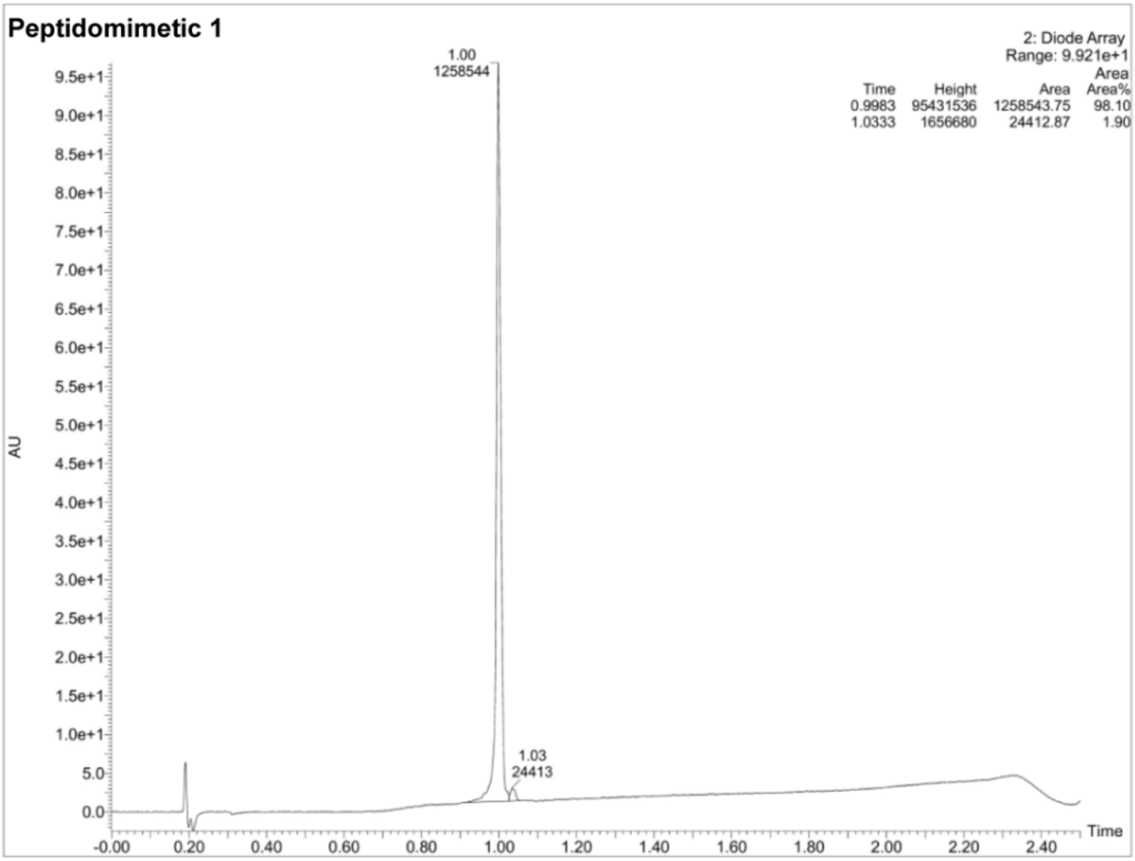

Time	Height	Area	Area%
0.9983	95431536	1258543.75	98.10
1.0333	1656680	24412.87	1.90

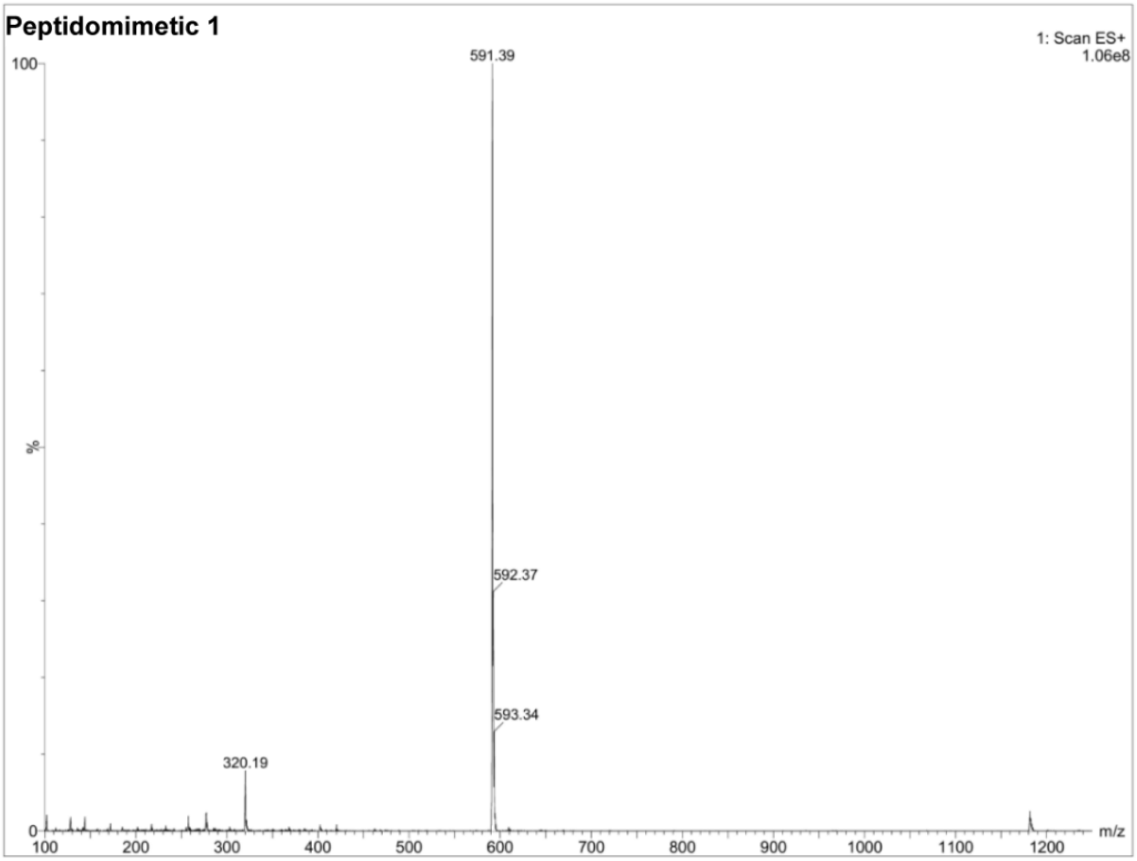

Supplementary Figure 11. UPLC chromatogram and MS of peptidomimetic **1**.

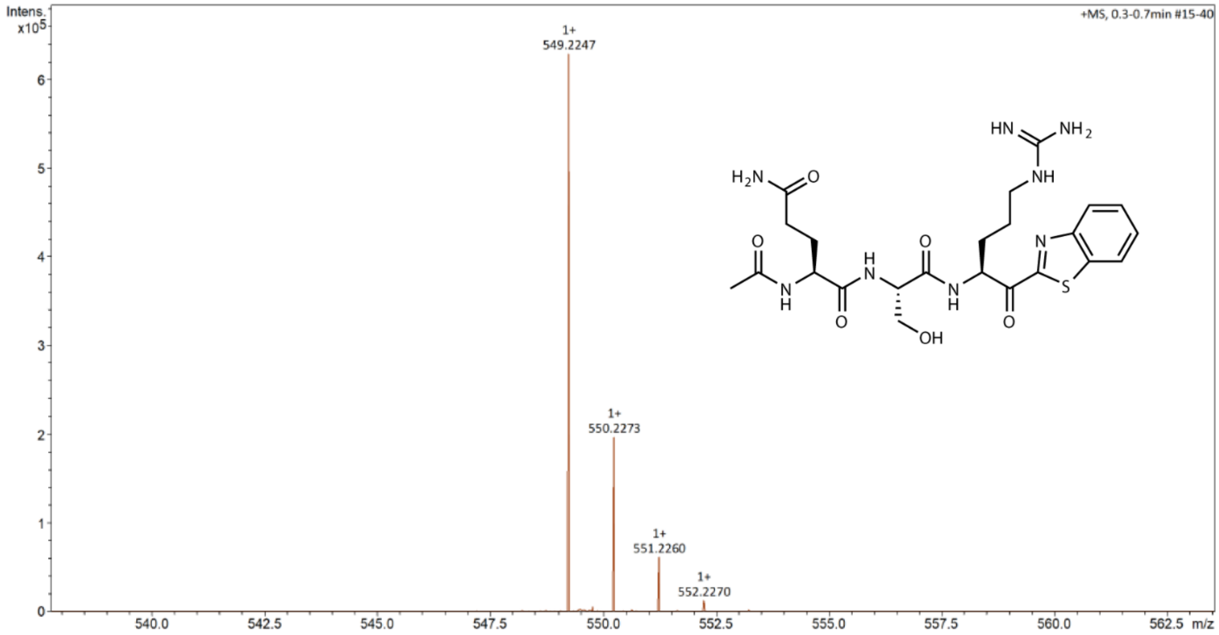

Supplementary Figure 12. Isotopic profile for the most abundant ion (singly charged) peptidomimetic **2**, $[M+H]^+$ detected with high-resolution mass spectrometry (Qtof).

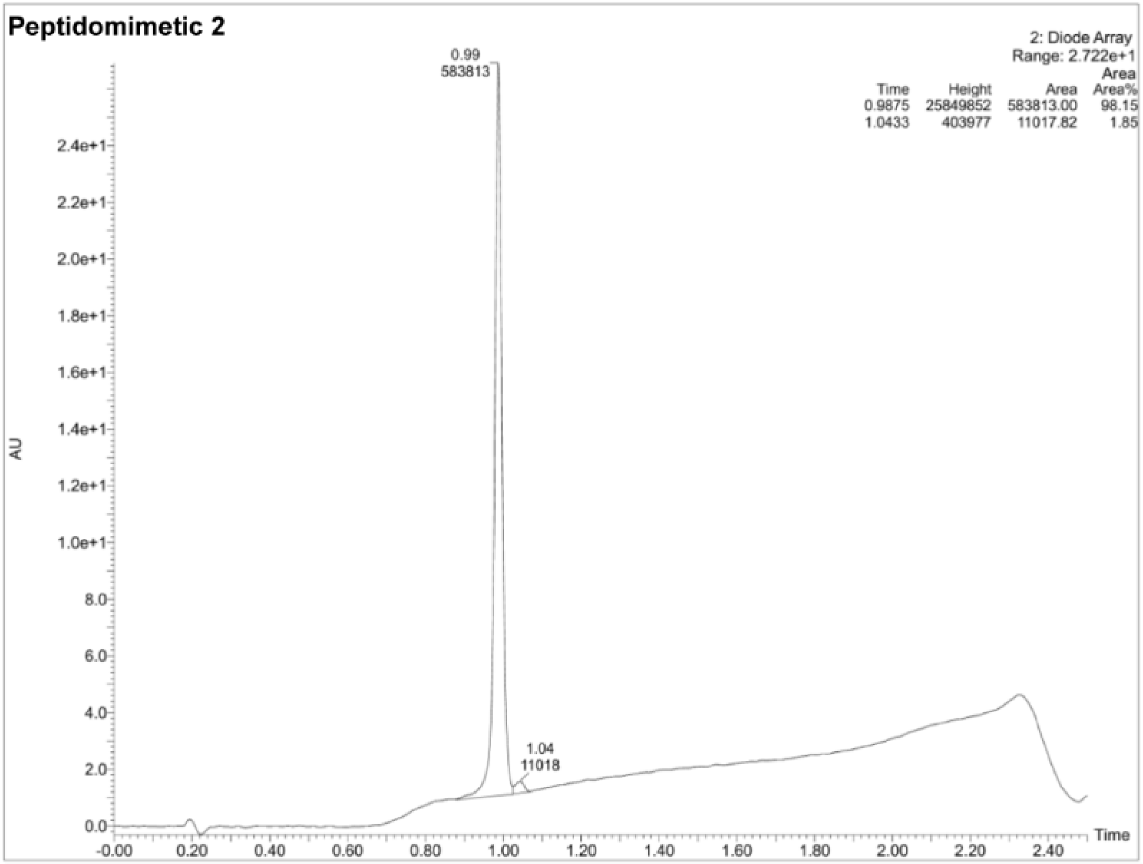
Peptidomimetic 2
2: Diode Array
Range: 2.722e+1
Time Height Area Area%
0.9875 25849852 583813.00 98.15
1.0433 403977 11017.82 1.85
0.99
583813
1.04
11018
AU
Time

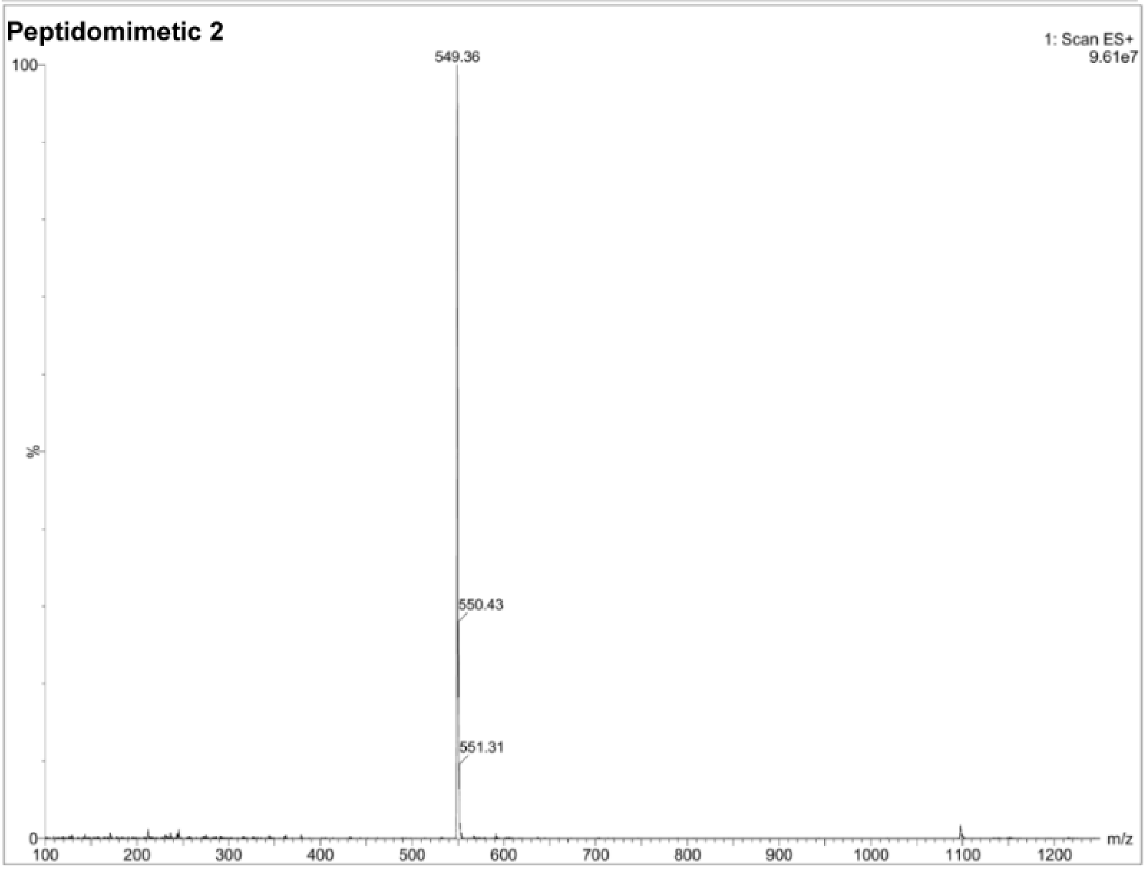
Peptidomimetic 2
1: Scan ES+
9.61e7
549.36
550.43
551.31
%
m/z

Supplementary Figure 13. UPLC chromatogram and MS of peptidomimetic **2**.

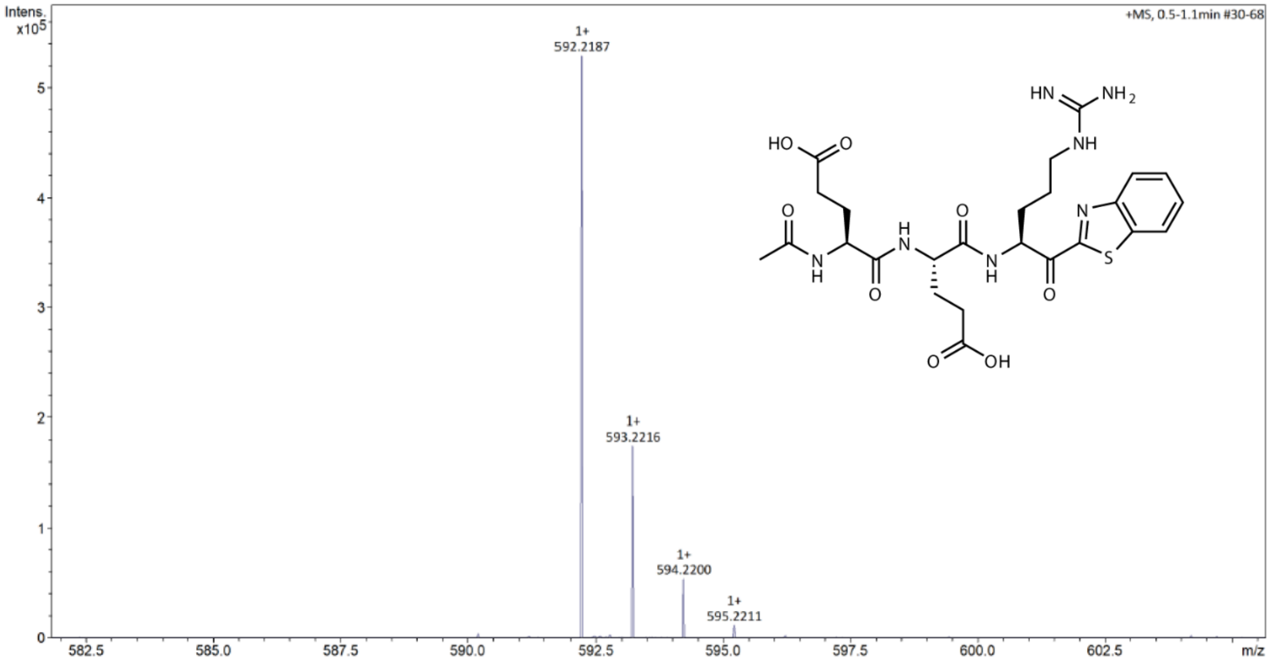

Supplementary Figure 14. Isotopic profile for the most abundant ion (singly charged) peptidomimetic **3**, $[M+H]^+$ detected with high-resolution mass spectrometry (Qtof).

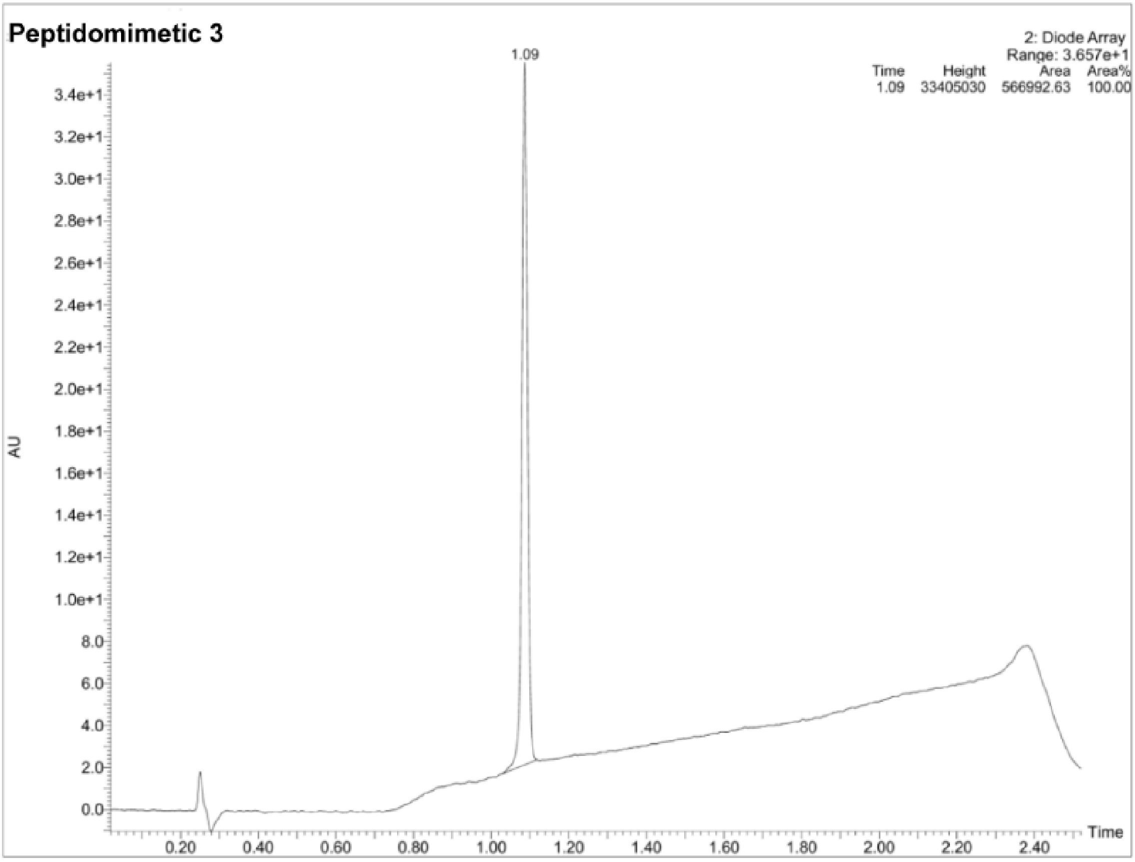
Peptidomimetic 3
2: Diode Array
Range: 3.657e+1
Time Height Area Area%
1.09 33405030 566992.63 100.00
1.09
AU
Time

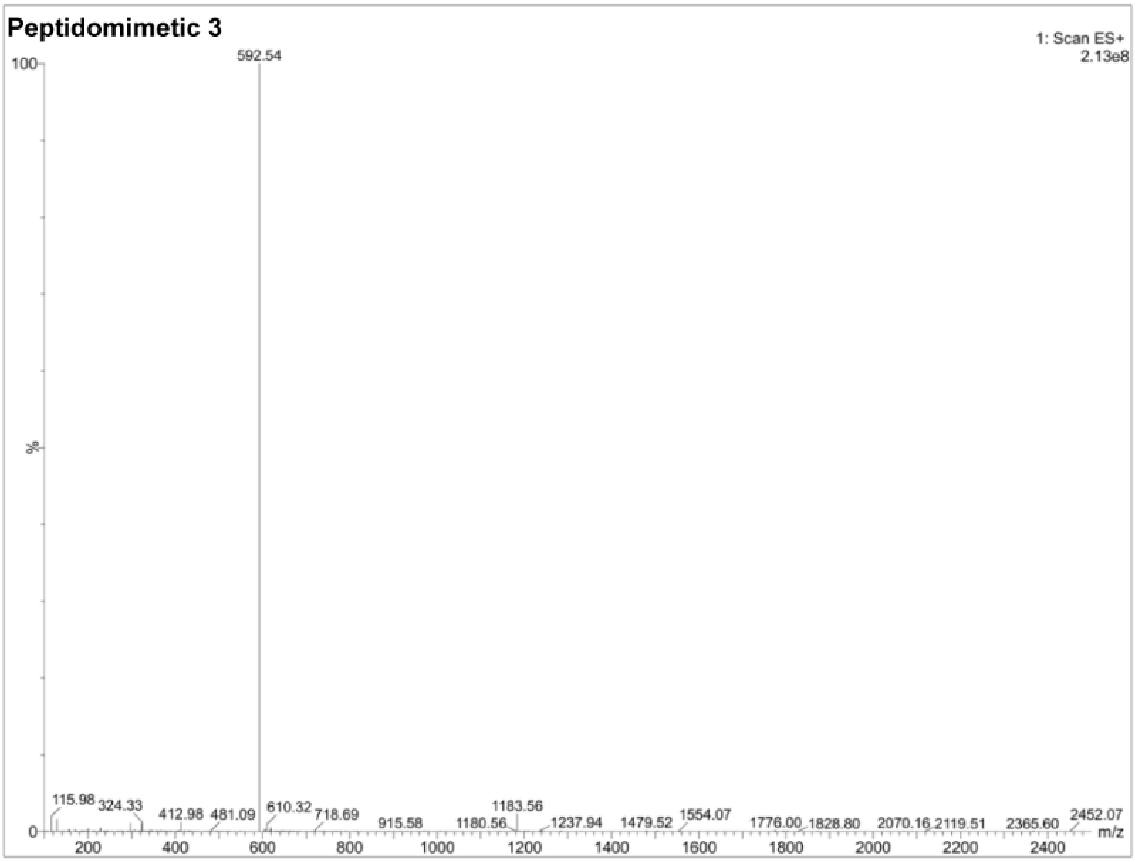
Peptidomimetic 3
1: Scan ES+
2.13e8
592.54
%
m/z

Supplementary Figure 15. UPLC chromatogram and MS of peptidomimetic **3**.

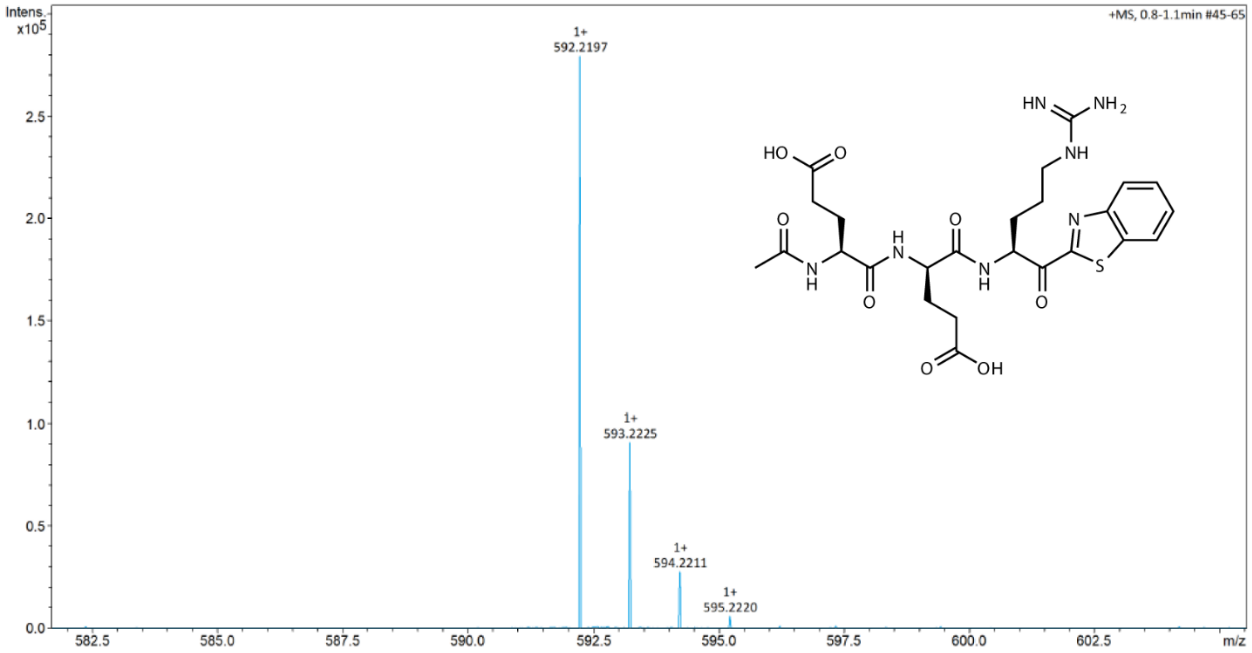

Supplementary Figure 16. Isotopic profile for the most abundant ion (singly charged) peptidomimetic **4**, $[M+H]^+$ detected with high-resolution mass spectrometry (Qtof).

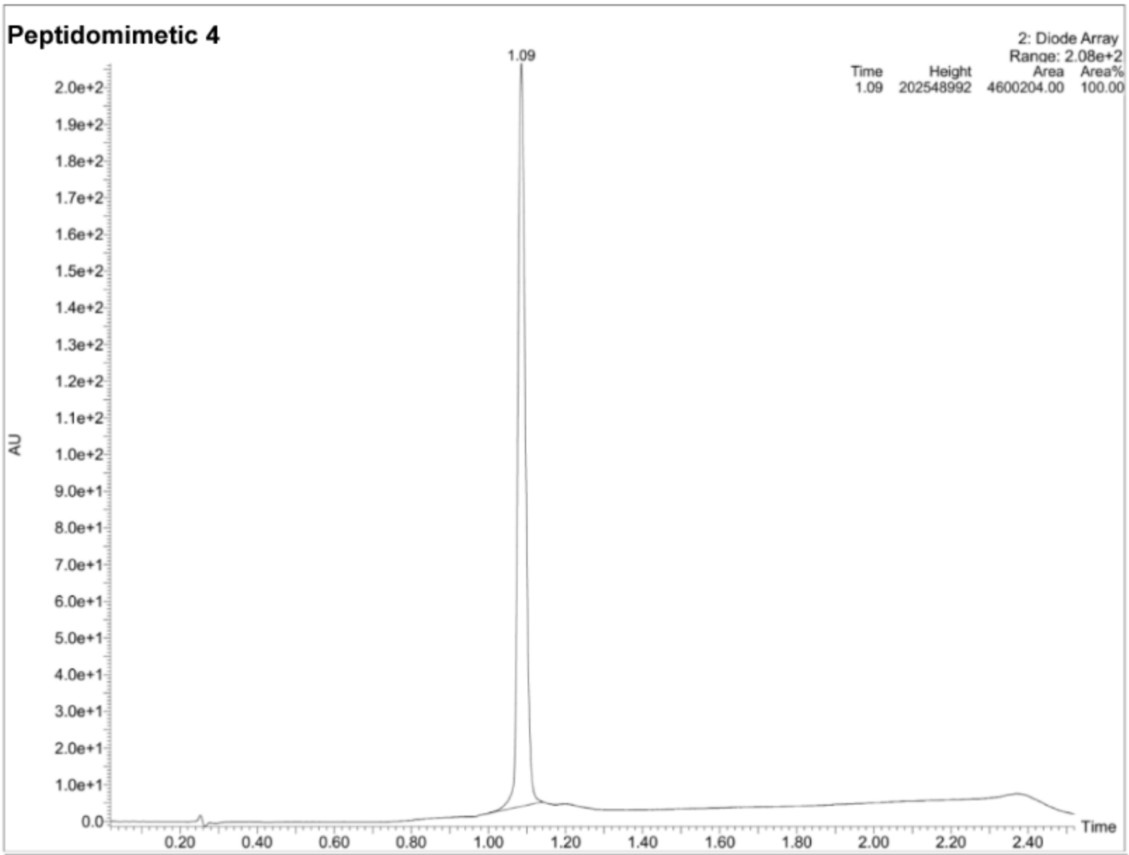
Peptidomimetic 4
2: Diode Array
Range: 2.08e+2
Time Height Area Area%
1.09 202548992 4600204.00 100.00
1.09
AU
Time

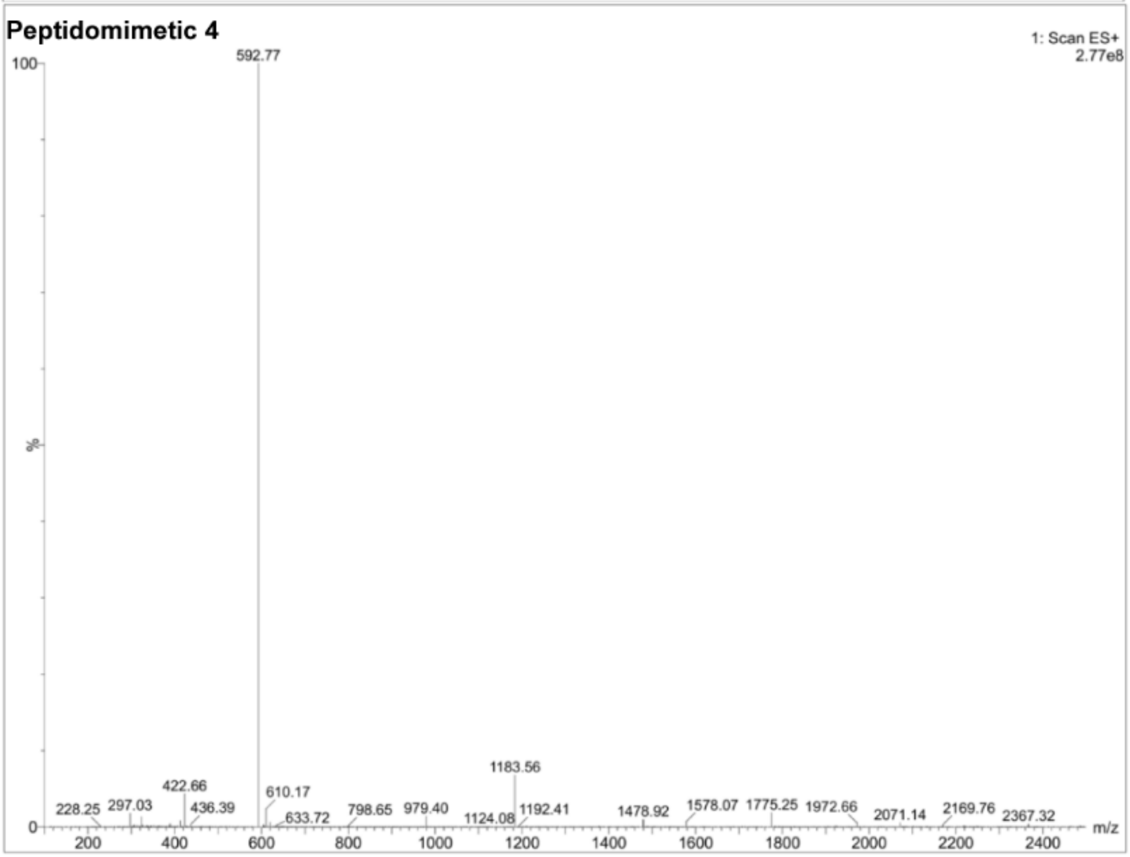
Peptidomimetic 4
1: Scan ES+
2.77e8
592.77
1183.56
422.66
610.17
228.25 297.03 436.39
633.72 798.65 979.40 1124.08 1192.41 1478.92 1578.07 1775.25 1972.66 2071.14 2169.76 2367.32
%
m/z

Supplementary Figure 17. UPLC chromatogram and MS of peptidomimetic **4**.

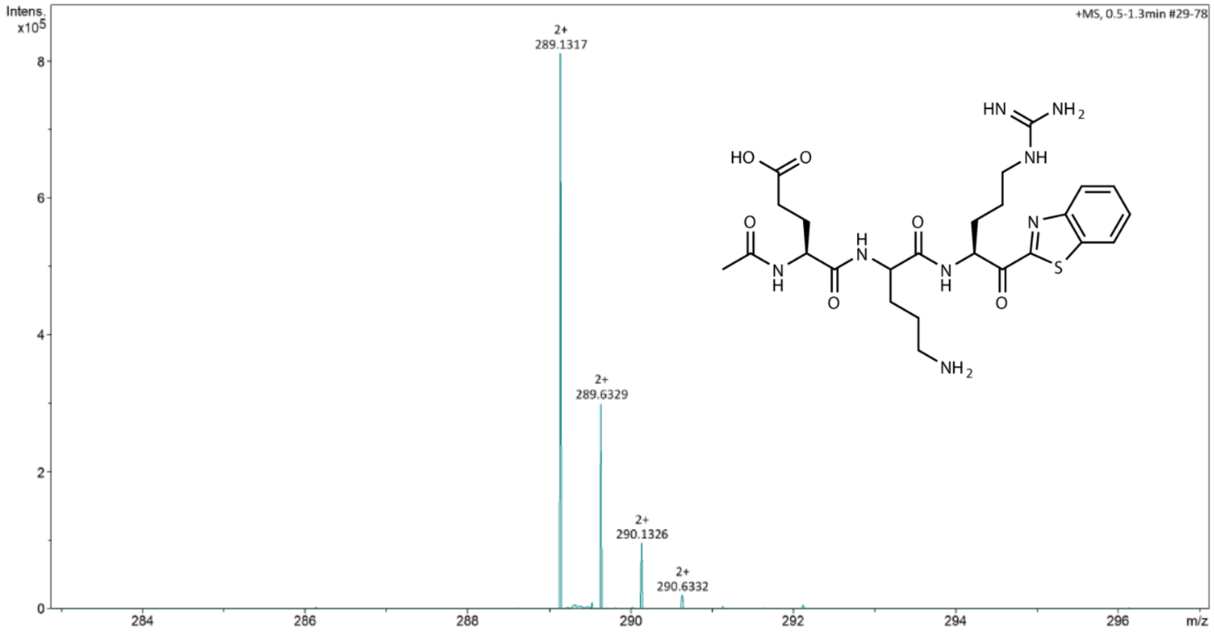

Supplementary Figure 18. Isotopic profile for the most abundant ion (doubly charged) peptidomimetic **4**, $[M+2H]^{2+}$ detected with high-resolution mass spectrometry (Qtof).

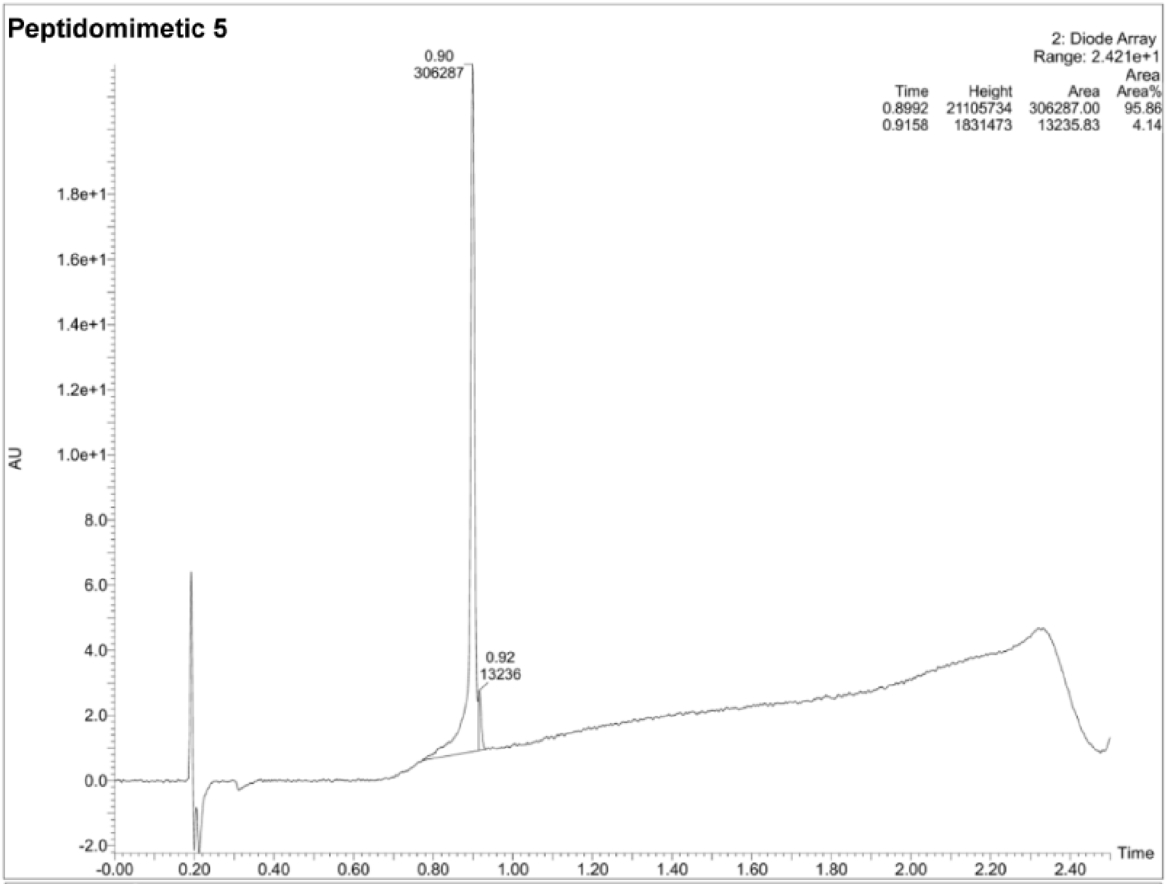
Peptidomimetic 5
2: Diode Array
Range: 2.421e+1
Time Height Area Area%
0.8992 21105734 306287.00 95.86
0.9158 1831473 13235.83 4.14
0.90
306287
0.92
13236
AU
Time

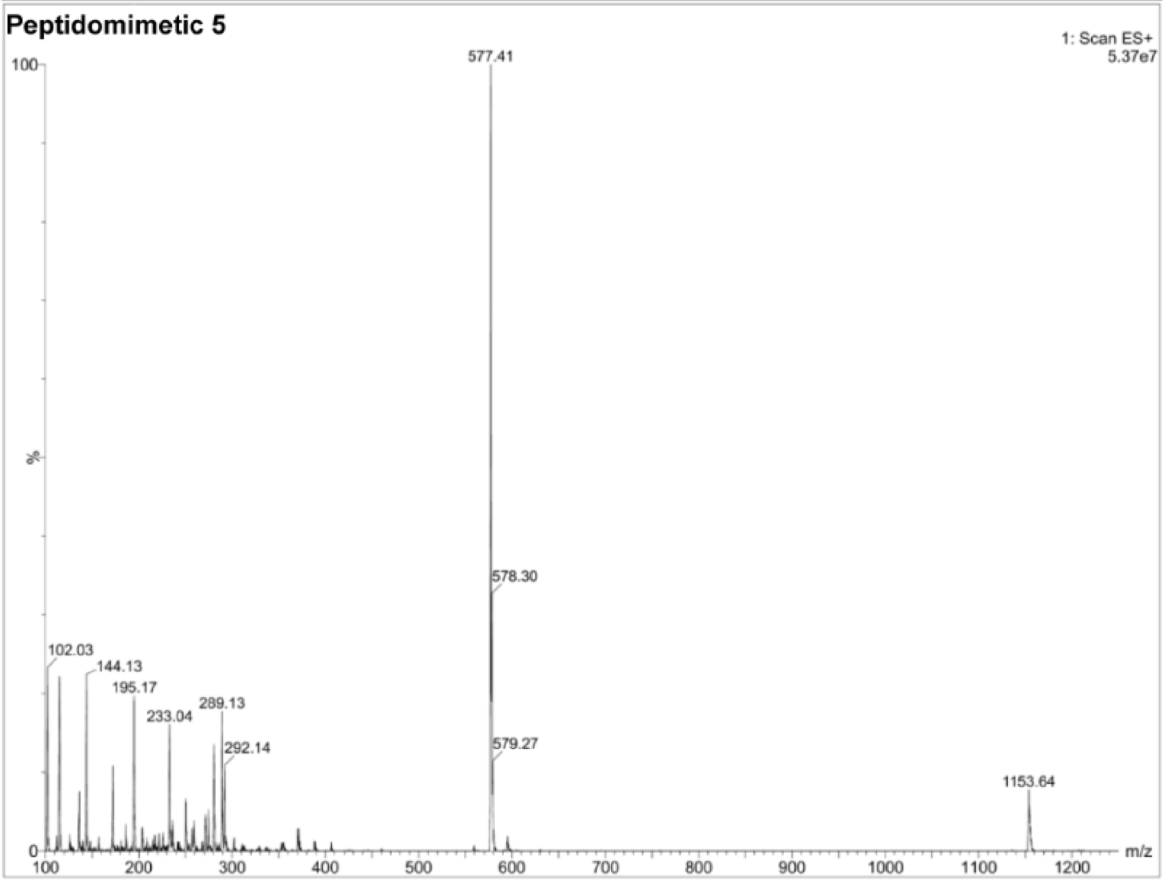
Peptidomimetic 5
1: Scan ES+
5.37e7
577.41
578.30
579.27
102.03
144.13
195.17
233.04
289.13
292.14
1153.64
%
m/z

Supplementary Figure 19. UPLC chromatogram and MS of peptidomimetic **5**.

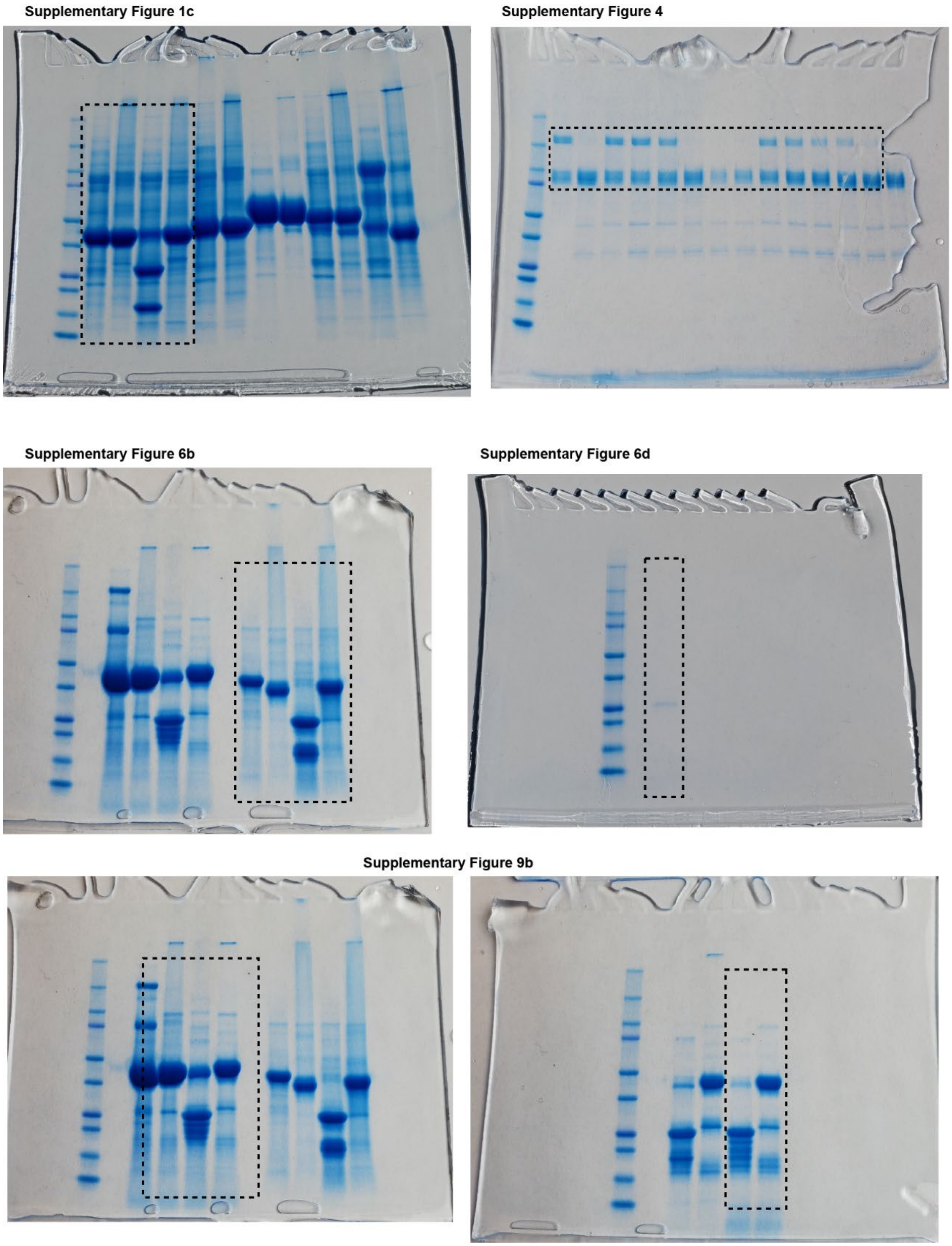

Supplementary Figure 20. Uncropped protein gel images for the indicated Supplementary Figure items.

Supplementary Table 1. PCR primers used for ligand-independent cloning and mutagenesis of TMPRSS11D constructs in this study.

TMPRSS11D construct	**Fwd_ Rev Primer- PCR-set1 sequence (5' to 3')**	**Fwd_ Rev Primer- PCR-set2 sequence (5' to 3')**	**Fwd_ Rev Primer- PCR-set3 sequence (5' to 3')**
eTMPRSS11D	tacatctatgcggccgctgatcaaaaatct tacttttataggag_ ggtgctcgacgaattcgatcccagtttgtt gcctaatc	x	x
eTMPRSS11D S368A	ttgtatttccagggcgatcaaaaatct tacttttataggag_ gccaccagcgtcaccctgacatgcg tcc	ggtgacgctggtggcccactagtaca agaagac _caagcttcgtcatcagatcccagttt gttgcctaatc	x
dasTMPRSS11D	tacatctatgcggccgctgatcaaaaatct tacttttataggag_ cttgtcgtcgtcgtctgttattaggtctg gacc	gacgacgacgacaagatccttggagg cactgagg_ggtgctcgacgaattcgat cccagtttgttgcctaatc	x
dasTMPRSS11D S368A	ttgtatttccagggcgatcaaaaatct tacttttataggag_ cttgtcgtcgtcgtctgttattaggtctg gacc	gacgacgacgacaagatccttggagg cactgagg_ gccaccagcgtcaccctgacatgcgt cc	ggtgacgctggtggcccactagtacaagaag ac_caagcttcgtcatcagatcccagtttgttg cctaatc

Supplementary Table 2. Summary of peptidomimetic compounds in this study, their expected and experimentally determined masses, and purity.

Name	Sequence	Formula	Mass (Da)			Purity %
			Calculated	UPLC/ MS $[M+H]^+$	HRMS $[M+H]^+$ or $[M+2H]^{2+}$	
**Peptidomimetic 1**	Ac-Glu-Gln-Arg-kbt	$C_{25}H_{34}N_8O_7S$	590.65	591.39	591.2346	98.10
**Peptidomimetic 2**	Ac-Gln-Ser-Arg-kbt	$C_{23}H_{32}N_8O_6S$	548.62	549.36	549.2247	98.15
**Peptidomimetic 3**	Ac-Glu-Glu-Arg-kbt	$C_{25}H_{33}N_7O_8S$	591.64	592.54	592.2187	100
**Peptidomimetic 4**	Ac-Glu-dGlu-Arg-kbt	$C_{25}H_{33}N_7O_8S$	591.64	592.77	592.2197	100
**Peptidomimetic 5**	Ac-Glu-Orn-Arg-kbt	$C_{25}H_{36}N_8O_6S$	576.67	577.41	289.1317	95.86

Supplementary Table 3. Recombinant dasTMPRSS11D and dasTMPRSS11D S368A protein melt temperature shifts in the presence of nafamostat and 6-amidino-2-naphthol.

dasTMPRSS11D

T_m = 60.5°C	Nafamostat (100 µM)	6-amidino-2-naphthol (10 mM)
T_m shift (°C)	9.2	4.7

dasTMPRSS11D S368A

T_m = 60.8°C	Nafamostat (100 µM)	6-amidino-2-naphthol (10 mM)
T_m shift (°C)	-0.7	2.17

Supplementary Table 4. Data collection and refinement statistics for TMPRSS11D protein crystal structures

	dasTMPRSS11D (PDB 8VIS)	eTMPRSS11D S368A (PDB 9DPF)
**Data collection**		
Space group	$P4_32_12$	$P2_12_12_1$
Cell dimensions		
a, *b*, *c* (Å)	119.68, 119.68, 134.29	73.03, 76.64, 88.37
α, β, γ (°)	90.00, 90.00, 90.00	90.00, 90.00, 90.00
Resolution (Å)	1.59(49.77-1.59) *	1.90(40.0-1.90)*
R_{merge}	0.23	0.22
$I / \sigma I$	1.57(1.59)	1.60 (1.91)
Completeness (%)	100.0 (49.77-1.59)	99.2 (38.31-1.90)
Redundancy	4.8 (4.3)	11.4 (9.4)
**Refinement**		
Resolution (Å)	1.59 (49.77-1.59)	1.90 (38.31-1.90)
No. reflections	129049	37404
R_{work} / R_{free}	0.155 / 0.187	0.202 / 0.257
No. atoms		
Protein	7732	3780
Ligand/ion	28	8
Water	1149	265
B-factors	17.0	34.0
Protein	14	32
Ligand/ion	20	59
Water	46.3	42.4
R.m.s. deviations		
Bond lengths (Å)	0.55	0.47
Bond angles (°)	0.89	0.77

*Each structure was solved using diffraction data from a single crystal. **Values in parentheses are for highest-resolution shell.

Supplementary Methods

Tripeptide synthesis

Solid-phase Peptide Synthesis (SPPS) was carried out either in 30–60 mL polypropylene cartridges equipped with sintered Teflon taps from Applied Separations (Allentown, PA, USA) or in 50–100 mL glass reactors from Chemglass Life Sciences (Vineland, NJ, USA).

Peptides were synthesized using solid-phase peptide synthesis on 2-chlorotrityl chloride resin (100-200 mesh dry, 0.23 mmol/g) using standard Fmoc chemistry. Coupling of Fmoc-protected amino acids (5.0 equiv) was achieved by treatment with hexafluorophosphate azabenzotriazole tetramethyluronium (HATU, 5.0 equiv) and DIPEA (10.0 equiv) at room temperature for 45 min. Capping of the resin was performed after the first amino acid coupling, to limit the unreacted sites on the resin, with 3 mL of a 7/2/1 mixture of DCM/MeOH/DIPEA for 20 min. The Fmoc-protecting group was removed by filtration upon mixing the resin with a solution of 20% piperidine in DMF for 10 min. This step was repeated twice. After each coupling and deprotection step, the resin was filtered thoroughly and washed with DMF (3 x 5 mL), DCM (3 x 5 mL), iPrOH (1 x 2 mL) and DMF (3 x 5 mL), using approximately 10 mL per gram of resin. Upon completing the linear sequence, the resin was dried under a stream of pressurized air. Then, a small amount of resin was cleaved with TFA for 10 min, and the peptide was analyzed by UPLC/MS.

N-terminal acetylation of tripeptide

After structure confirmation by UPLC-MS, the Fmoc-protecting group was removed by filtration using a solution of 20% piperidine in DMF for 10 min. This step was repeated twice and was followed by resin washing with DMF (3 x 5 mL), DCM (3 x 5 mL), iPrOH (1 x 2 mL) and DCM (3 x 5 mL). Then, all peptides were treated on resin with acetic anhydride (3 equiv) and DIPEA (4.5 equiv) in DCM (5 mL for 0.1 mmol of resin) for 20 minutes on an orbital shaker at 120 rpm. Prior to peptide cleavage, the resin was washed with DCM (3 x 5 mL).

Cleavage from the resin

Peptides were cleaved from the resin using a 30% solution of hexafluoroisopropanol (HFIP) in DCM (3 mL per 0.1 mmol of resin) while shaken on an orbital shaker at 120 rpm for 1 h. The mixture was subsequently filtered, and the solution was evaporated *in vacuo* on rotary evaporator. This step was repeated once more for 30 min using the same cleavage solution, followed by a thorough wash of the resin with DCM (10 mL/g of resin). The cleaved resin was discarded and the solution was evaporated *in vacuo* on rotary evaporator once again. The remaining acidic solution not eliminated on rotary evaporator was evaporated using a SpeedVac Savant SC250EXP concentrator (SpectraLab Scientific Inc., Markham, ON, Canada). The crude peptide was purified by preparative HPLC-UV/Visible system.

Crude peptide purification

The crude peptide intermediates were dissolved in a 10 mL mixture of acetic acid and H_2O (ratio dependent on peptide solubility) and filtered through a 0.22 µm PTFE Chromatography Syringe Filter. The peptides were then purified using Waters preparative HPLC-UV/Visible system (column ACMP-10-25030P – 30 x 250 mm, packed with 10 µm particles; UV/Visible detector 2489; Waters 2707 Autosampler; and Waters Fraction Collector III). The purified intermediates were lyophilized overnight in a freeze dryer (LABCONCO, Kansas City, MO, USA).

Coupling of the warhead

The previously reported warhead[1] NH_2-Arg(Pbf)-Kbt (1.0 equiv), was coupled with purified intermediates (1.2 equiv) bearing standard protection groups using HATU (1.2 equiv) and DIPEA (3.0 equiv) in DMF. The reaction was agitated for 4 h at room temperature or longer until complete conversion, as confirmed by UPLC-MS. Then, the DMF was evaporated under a stream of pressurized air for 2 days. To the resulting orange crude, a water-immiscible organic solvent (e.g., ethyl acetate (EtOAc)) was added. The organic layer was sequentially washed with saturated sodium bicarbonate ($NaHCO_3$) and brine to remove impurities. The organic layer was dried over anhydrous sodium sulfate (Na_2SO_4) and filtered to remove the drying agent, then the organic solvent was evaporated *in vacuo* on rotary evaporator.

Oxidation of alcohol moiety for serine trap obtention

To all precursor compounds solubilized in DCM, Dess-Martin periodinane (DMP, 1.5 equiv) was added slowly at 0 °C and the resulting solution was stirred for 10 min at 0 °C. The cooling bath was removed and the reaction solution was stirred for an additional 30 min at room temperature. Then, a saturated aqueous solution of $Na_2S_2O_3$ was added and let to stir until the complete disappearance of the milky DMP suspension. This step was repeated twice if the precipitate did not disappear after 30 min. Then, the supernatant was removed, and the resulting solution was washed with sat. aqueous $NaHCO_3$ (20mL), brine (20mL), dried over anhydrous Na_2SO_4, filtered and concentrated *in vacuo*.

Side chain deprotection of acid-labile groups

To the crude product, a solution of TFA/TIPS/H_2O (95/2.5/2.5) was added, and the reaction mixture was stirred for 2 h on an orbital shaker at 120 rpm. The remaining acidic solution was evaporated, and purification was carried out on the same day.

Final purification of peptidomimetics

The crude compounds were dissolved in a 10 mL mixture of acetic acid and H_2O depending on peptide solubility, and filtered through a 0.22 μm PTFE Chromatography Syringe Filter. The peptides were then purified using a Waters preparative HPLC-UV/Visible system (column ACMP-10-25030P – 30 x 250 mm, packed with 10 μm particles; UV/Visible detector 2489; Waters 2707 Autosampler; and Waters Fraction Collector III). The purified intermediates were lyophilized overnight in a freeze dryer (LABCONCO, Kansas City, MO, USA).

Only the predominant diastereoisomer was isolated with a purity exceeding 95% and used in subsequent experiments.

DynaFit 4.10.004 scripts for microscopic rate constant determination

TMPRSS11D kinetic data was formatted for input in the DynaFit 4 software package (input data is available in the Source Data document) and curve-fitted using scripts described in the following sections. Scripts were designed using covalent inhibitor script templates available in the DynaFit software package and using the DynaFit scripting manual (v4.08.005)[1,2]. Experiments were designed to measure the acylation and de-acylation phases of nafamostat, and competitive inhibition by 6-amidino-2-naphthol. All input data and script files describe concentrations in micromolar (μM) and reaction progress time in seconds (s).

A) TMPRSS11D k_{sub} determination for Boc-QAR-AMC substrate

```
[task]
  data = progress
  task = fit
[mechanism]
  E + S --> E + P   :     ksub
[constants]
  ksub = 0.25?
[concentrations]
  E = 0.0010?
  S = 100
[responses]
  P = 1
[data]
directory ./TMPRSS11D/input
  sheet     11D_ksub_May25.csv
  monitor E, S, E.S, P
  column   2 | offset = 0?   | conc E = 0.0025?   | label E=2.5 nM
  column   3 | offset = 0?   | conc E = 0.0025?   | label E=2.5 nM
  column   4 | offset = 0?   | conc E = 0.0025?   | label E=2.5 nM
  column   5 | offset = 0?   | conc E = 0.0025?   | label E=2.5 nM
  column   6 | offset = 0?   | conc E = 0.005?   | label E=5 nM
  column   7 | offset = 0?   | conc E = 0.005?   | label E=5 nM
  column   8 | offset = 0?   | conc E = 0.005?   | label E=5 nM
  column   9 | offset = 0?   | conc E = 0.005?   | label E=5 nM
  column   10 | offset = 0?   | conc E = 0.010?   | label E=10 nM
  column   11 | offset = 0?   | conc E = 0.010?   | label E=10 nM

[output]
directory ./TMPRSS11D/output/ksub_calibration_2
[settings]
{Filter}
  TimeMin = 1
  ZeroBaselineSignal = y
{Output}
  XAxisLabel = time, sec
  YAxisLabel = product, uM
[end]
```

B) TMPRSS11D k_{inact}/K_I determination for nafamostat

```
[task]
  data = progress
  task = fit
[mechanism]
  E + S --> E + P   :     ksub
  E + I --> E-I     :     keff
[constants]
  ksub = 0.049
  keff = 0.1?
[concentrations]
  E = 0.003
  S = 100
[responses]
  P = 1
[data]
directory ./TMPRSS11D/input
  sheet     11D_naf_May25.csv
  monitor E, E.S, E.I, E-I,
  column   2 | offset = 0?   | conc I = 1.6   | label I=1600 nM
  column   3 | offset = 0?   | conc I = 1.6   | label I=1600 nM
  column   4 | offset = 0?   | conc I = 0.8   | label I=800 nM
  column   5 | offset = 0?   | conc I = 0.8   | label I=800 nM
  column   6 | offset = 0?   | conc I = 0.4   | label I=400 nM
  column   7 | offset = 0?   | conc I = 0.4   | label I=400 nM
  column   8 | offset = 0?   | conc I = 0.2   | label I=200 nM
  column   9 | offset = 0?   | conc I = 0.2   | label I=200 nM
  column   10 | offset = 0?   | conc I = 0.1   | label I=100 nM
  column   11 | offset = 0?   | conc I = 0.1   | label I=100 nM
  column   12 | offset = 0?  | conc I = 0.05   | label I=50 nM
  column   13 | offset = 0?   | conc I = 0.05   | label I=50 nM
  column   14 | offset = 0?   | conc I = 0.025   | label I=25 nM
  column   15 | offset = 0?   | conc I = 0.025   | label I=25 nM
  column   16 | offset = 0?   | conc I = 0.0125   | label I=12.5 nM
  column   17 | offset = 0?   | conc I = 0.0125   | label I=12.5 nM
  column   18 | offset = 0?   | conc I = 0.0063   | label I=6.3 nM
  column   19 | offset = 0?   | conc I = 0.0063   | label I=6.3 nM
  column   20 | offset = 0?   | conc I = 0.0031   | label I=3.1 nM
  column   21 | offset = 0?   | conc I = 0.0031   | label I=3.1 nM
  column   22 | offset = 0?   | conc I = 0.0016   | label I=1.6 nM
  column   23 | offset = 0?   | conc I = 0.0016   | label I=1.6 nM
  column   24 | offset = 0?   | conc I = 0.0008   | label I=0.8 nM
  column   25 | offset = 0?   | conc I = 0.0008   | label I=0.8 nM
  column   26 | offset = 0?   | conc I = 0.0   | label I=0 nM
  column   27 | offset = 0?   | conc I = 0.0   | label I=0 nM
  column   28 | offset = 0?   | conc I = 0.0   | label I=0 nM
```

```
  column   29 | offset = 0?   | conc I = 0.0   | label I=0 nM
[output]
directory ./TMPRSS11D/Output/nafamostat_May26
[settings]
{Filter}
  TimeMin = 1
  ZeroBaselineSignal = y
{Output}
  XAxisLabel = time, sec
  YAxisLabel = product, uM
```

C) TMPRSS11D $k_{hydrolysis}$ and $t_{1/2}$ determination for nafamostat

```
[task]
  data = progress
  task = fit
[mechanism]
E.I   --> E + I'   : khydrolysis
E + S --> E + P    : ksub
E + I --> E.I      : keff

[constants]
  ksub = 0.049?
  khydrolysis= 10?
  keff = 0.001566

[concentrations]
  E.I = 0.0084
  S = 83
[responses]
  P = 1

[data]
directory ./TMPRSS11D/input
  sheet     11D_naf_reversibility_v2.csv
  monitor E, S, E.S, P
  column   2 | offset = 0?   | conc I = 1   | label I=1000 nM
  column   3 | offset = 0?  | conc I = 1   | label I=1000 nM
  column   4 | offset = 0?   | conc I = 0.5   | label I=500 nM
  column   5 | offset = 0?   | conc I = 0.5   | label I=500 nM

[output]
directory ./TMPRSS11D/output/naf_reversibility_Oct7_revised
[settings]
{Filter}
  TimeMin = 1
  ZeroBaselineSignal = y
{Output}
  XAxisLabel = time, sec
  YAxisLabel = product, uM
[end]
```

D) TMPRSS11D K_i determination for 6-amidino-2-naphthol

```
[task]
  data = progress
  task = fit
[mechanism]
  E + S --> E + P   :     ksub
  E + I --> E.I     :     k.a
  E.I --> E + I     :     k.d
[constants]
  ksub = 0.049
  k.a = 1
  k.d = 10?
[concentrations]
  E = 0.0031
  S = 100
[responses]
  P = 1
[data]
directory ./TMPRSS11D/input
  sheet     11D_6am_May25_revised.csv
  monitor E, E.S, E.I, E-I,
  column   2 | offset = 0?   | conc I = 500   | label I=500 uM
  column   3 | offset = 0?   | conc I = 500   | label I=500 uM
  column   4 | offset = 0?   | conc I = 250   | label I=250 uM
  column   5 | offset = 0?   | conc I = 250   | label I=250 uM
  column   6 | offset = 0?   | conc I = 125   | label I=125 uM
  column   7 | offset = 0?   | conc I = 125   | label I=125 uM
  column   8 | offset = 0?   | conc I = 63   | label I=63 uM
  column   9 | offset = 0?   | conc I = 63   | label I=63 uM
  column   10 | offset = 0?   | conc I = 31   | label I=31 uM
  column   11 | offset = 0?   | conc I = 31   | label I=31 uM
  column   12 | offset = 0? | conc I = 16   | label I=16 uM
  column   13 | offset = 0?   | conc I = 16   | label I=16 uM
  column   14 | offset = 0?   | conc I = 8   | label I=8 uM
  column   15 | offset = 0?   | conc I = 8   | label I=8 uM
  column   16 | offset = 0?   | conc I = 4   | label I=4 uM
  column   17 | offset = 0?   | conc I = 4   | label I=4 uM
  column   18 | offset = 0?   | conc I = 2   | label I=2 uM
  column   19 | offset = 0?   | conc I = 2   | label I=2 uM
  column   20 | offset = 0?   | conc I = 1   | label I=1 uM
  column   21 | offset = 0?   | conc I = 1   | label I=1 uM
  column   22 | offset = 0?   | conc I = 0.5   | label I=0.5 uM
  column   23 | offset = 0?   | conc I = 0.5   | label I=0.5 uM
  column   24 | offset = 0?   | conc I = 0.25   | label I=0.25 uM
  column   25 | offset = 0?   | conc I = 0.25   | label I=0.25 uM
  column   26 | offset = 0?   | conc I = 0.0   | label I=0 nM
```

```
  column   27 | offset = 0?   | conc I = 0.0   | label I=0 nM
  column   28 | offset = 0?   | conc I = 0.0   | label I=0 nM
  column   29 | offset = 0?   | conc I = 0.0   | label I=0 nM
[output]
directory ./TMPRSS11D/Output/6amidino_revised_Oct7
[settings]
{Filter}
  TimeMin = 1
  ZeroBaselineSignal = y
{Output}
  XAxisLabel = time, sec
  YAxisLabel = product, uM
[end]
```

Supplementary References

1. Kuzmič, P. Deciding between one-step and two-step irreversible inhibition mechanisms on the basis of “kobs” data: A statistical approach. *bioRxiv* (2020) doi:10.1101/2020.06.08.140160.

2. Kuzmic, P. *DynaFit Scripting Software Version 4.08*. www.biokin.com/dynafit/manual.html (2018).
