# Supplementary material for: Structural basis of TMPRSS11D specificity and autocleavage activation
Source: Nat Commun. 2025 May 10;16:4351. doi: 10.1038/s41467-025-59677-3 (PMC12065894; doi:10.1038/s41467-025-59677-3)
Supplement: Supplementary file 2 — Reporting Summary [file 41467_2025_59677_MOESM2_ESM.pdf]

nature portfolio

Corresponding author(s): Cheryl H. Arrowsmith, Francois Benard, Gregg B. Morin, Bryan J. Fraser

Last updated by author(s): Apr 21, 2025

Reporting Summary

Nature Portfolio wishes to improve the reproducibility of the work that we publish. This form provides structure for consistency and transparency in reporting. For further information on Nature Portfolio policies, see our Editorial Policies and the Editorial Policy Checklist.

Statistics

For all statistical analyses, confirm that the following items are present in the figure legend, table legend, main text, or Methods section.

n/a	Confirmed	
☐	☒	The exact sample size (*n*) for each experimental group/condition, given as a discrete number and unit of measurement
☐	☒	A statement on whether measurements were taken from distinct samples or whether the same sample was measured repeatedly
☐	☒	The statistical test(s) used AND whether they are one- or two-sided<br>*Only common tests should be described solely by name; describe more complex techniques in the Methods section.*
☒	☐	A description of all covariates tested
☒	☐	A description of any assumptions or corrections, such as tests of normality and adjustment for multiple comparisons
☐	☒	A full description of the statistical parameters including central tendency (e.g. means) or other basic estimates (e.g. regression coefficient) AND variation (e.g. standard deviation) or associated estimates of uncertainty (e.g. confidence intervals)
☐	☒	For null hypothesis testing, the test statistic (e.g. *F*, *t*, *r*) with confidence intervals, effect sizes, degrees of freedom and *P* value noted<br>*Give P values as exact values whenever suitable.*
☒	☐	For Bayesian analysis, information on the choice of priors and Markov chain Monte Carlo settings
☒	☐	For hierarchical and complex designs, identification of the appropriate level for tests and full reporting of outcomes
☒	☐	Estimates of effect sizes (e.g. Cohen's *d*, Pearson's *r*), indicating how they were calculated

Our web collection on statistics for biologists contains articles on many of the points above.

Software and code

Policy information about availability of computer code

Data collection	X-ray diffraction data were collected on the CMCF-ID beamline at the Canada Light Source (CLS). Enzyme kinetics were monitored using fluorescent reads on a BioTek Synergy H1 plate reader or on a Molecular Devices FlexStation 3 plate reader using the Gen5 (v3.03) and SoftMax Pro (v5.4.6) software packages, respectively. Differential Scanning Fluorimetry (DSF) data was collected on the Light Cycler 480 II (Roche Applied Sciences). Peptidomimetic compound masses were determined using a maXis 3G orthogonal mass spectrometer (ESI-QqTOFMS) (Bruker Daltonik; Bremen, Germany) using electrospray ionization in positive (or negative) ion mode.
Data analysis	X-ray diffraction data were processed with HKL-3000 (v720). Phaser MR (v2.8.3; CCP4Interface 8.0.006) was used for molecular replacement. Models were built with WinCOOT (v0.9.8.1 EL) and validated with MolProbity (v4.5.2). Enzyme kinetic data was curve-fitted using the DynaFit version 4.10.004 software package. Protein melting temperature data was processed using the DSFWorld online portal (https://gestwickilab.shinyapps.io/dsfworld/). TTSP protein sequences were aligned using Clustal Omega (https://www.ebi.ac.uk/jdispatcher/msa/clustalo). Manuscript data was plotted using GraphPad prism (v10.2.2). Protein structure images were generated using PyMOL (v2.5.7). Peptidomimetic compound MS spectra data were processed using Bruker Compass DataAnalysis 4.3.

For manuscripts utilizing custom algorithms or software that are central to the research but not yet described in published literature, software must be made available to editors and reviewers. We strongly encourage code deposition in a community repository (e.g. GitHub). See the Nature Portfolio guidelines for submitting code & software for further information.

Data

Policy information about availability of data

All manuscripts must include a data availability statement. This statement should provide the following information, where applicable:

- Accession codes, unique identifiers, or web links for publicly available datasets
- A description of any restrictions on data availability
- For clinical datasets or third party data, please ensure that the statement adheres to our policy

The coordinates and structures of dasTMPRSS11D complexed with a DDDDK peptide and eTMPRSS11D S368A complexed with a LSEQR peptide have been deposited to the PDB with accession numbers 8VIS and 9DPF, respectively. The engineered dasTMPRSS11D and eTMPRSS11D S368A protein expression constructs are available on Addgene (Plasmid nos. 220881 and 220882, respectively). Zymogen matriptase, active matriptase, TMPRSS2, bovine enteropeptidase, human enteropeptidase, TMPRSS11E, and TMPRSS13 structures were accessed using PDB IDs 5LY0, 4JYT, 8V04, 1EKB, 7WR7, 2OQ5, and 6KD5, respectively. All other relevant manuscript data are available in the source data provided with this paper.

Research involving human participants, their data, or biological material

Policy information about studies with human participants or human data. See also policy information about sex, gender (identity/presentation), and sexual orientation and race, ethnicity and racism.

Reporting on sex and gender	*Use the terms sex (biological attribute) and gender (shaped by social and cultural circumstances) carefully in order to avoid confusing both terms. Indicate if findings apply to only one sex or gender; describe whether sex and gender were considered in study design; whether sex and/or gender was determined based on self-reporting or assigned and methods used.*<br>*Provide in the source data disaggregated sex and gender data, where this information has been collected, and if consent has been obtained for sharing of individual-level data; provide overall numbers in this Reporting Summary. Please state if this information has not been collected.*<br>*Report sex- and gender-based analyses where performed, justify reasons for lack of sex- and gender-based analysis.*
Reporting on race, ethnicity, or other socially relevant groupings	*Please specify the socially constructed or socially relevant categorization variable(s) used in your manuscript and explain why they were used. Please note that such variables should not be used as proxies for other socially constructed/relevant variables (for example, race or ethnicity should not be used as a proxy for socioeconomic status).*<br>*Provide clear definitions of the relevant terms used, how they were provided (by the participants/respondents, the researchers, or third parties), and the method(s) used to classify people into the different categories (e.g. self-report, census or administrative data, social media data, etc.)*<br>*Please provide details about how you controlled for confounding variables in your analyses.*
Population characteristics	*Describe the covariate-relevant population characteristics of the human research participants (e.g. age, genotypic information, past and current diagnosis and treatment categories). If you filled out the behavioural & social sciences study design questions and have nothing to add here, write "See above."*
Recruitment	*Describe how participants were recruited. Outline any potential self-selection bias or other biases that may be present and how these are likely to impact results.*
Ethics oversight	*Identify the organization(s) that approved the study protocol.*

Note that full information on the approval of the study protocol must also be provided in the manuscript.

Field-specific reporting

Please select the one below that is the best fit for your research. If you are not sure, read the appropriate sections before making your selection.

[x] Life sciences [ ] Behavioural & social sciences [ ] Ecological, evolutionary & environmental sciences

For a reference copy of the document with all sections, see nature.com/documents/nr-reporting-summary-flat.pdf

Life sciences study design

All studies must disclose on these points even when the disclosure is negative.

Sample size	The number of technical replicates were sufficient to provide a degree of uncertainty of 20% or less and therefore judged to allow unambiguous conclusions to be drawn. Exact sample sizes and replicates are described in Method and figure legends.
Data exclusions	Single datapoint outliers are highlighted in Source Data Excel sheets and have been specifically called out in the Method section of the manuscript. Statistical tests for single datapoint outlier exclusions are outlined in the manuscript.
Replication	All kinetic measurements were reproducible within the uncertainties reported. In most cases, the experiments were successfully repeated on separate dates with at minimum n=3 independent biological experiments, with specific replicate details available in the manuscript.
Randomization	Kinetic measurements were performed as a series of independent experiments. Randomization has no meaning to the present work.

Blinding	Kinetic measurements were performed as a series of independent experiments, and, as repetitions of identical procedures, blinding has no meaning or relevance to the work.

Reporting for specific materials, systems and methods

We require information from authors about some types of materials, experimental systems and methods used in many studies. Here, indicate whether each material, system or method listed is relevant to your study. If you are not sure if a list item applies to your research, read the appropriate section before selecting a response.

Materials & experimental systems

n/a	Involved in the study
☒	☐ Antibodies
☐	☒ Eukaryotic cell lines
☒	☐ Palaeontology and archaeology
☒	☐ Animals and other organisms
☒	☐ Clinical data
☒	☐ Dual use research of concern
☒	☐ Plants

Methods

n/a	Involved in the study
☒	☐ ChIP-seq
☒	☐ Flow cytometry
☒	☐ MRI-based neuroimaging

Eukaryotic cell lines

Policy information about cell lines and Sex and Gender in Research

Cell line source(s)	Gibco™ Spodoptera frugiperda (Sf9) cells, Catalog # 12659027 (Thermo Fisher)
Authentication	Cell lines were tested for authentication by STR profiling
Mycoplasma contamination	All cell lines were mycoplasma negative, as determined by MycoAlert® Mycoplasma Detection Kit (Lonza Cat# LT07-318)
Commonly misidentified lines (See ICLAC register)	No commonly misidentified cell lines were used according to ICLAC database (http://iclac.org/databases/cross-contaminations/)

Plants

Seed stocks	*Report on the source of all seed stocks or other plant material used. If applicable, state the seed stock centre and catalogue number. If plant specimens were collected from the field, describe the collection location, date and sampling procedures.*
Novel plant genotypes	*Describe the methods by which all novel plant genotypes were produced. This includes those generated by transgenic approaches, gene editing, chemical/radiation-based mutagenesis and hybridization. For transgenic lines, describe the transformation method, the number of independent lines analyzed and the generation upon which experiments were performed. For gene-edited lines, describe the editor used, the endogenous sequence targeted for editing, the targeting guide RNA sequence (if applicable) and how the editor was applied.*
Authentication	*Describe any authentication procedures for each seed stock used or novel genotype generated. Describe any experiments used to assess the effect of a mutation and, where applicable, how potential secondary effects (e.g. second site T-DNA insertions, mosiacism, off-target gene editing) were examined.*
